# In Silico Investigation of Potential Applications of Gamma Carbonic Anhydrases as Catalysts of CO_2_ Biomineralization Processes: A Visit to the Thermophilic Bacteria *Persephonella hydrogeniphila, Persephonella marina, Thermosulfidibacter takaii,* and *Thermus thermophilus*

**DOI:** 10.3390/ijms22062861

**Published:** 2021-03-11

**Authors:** Colleen Varaidzo Manyumwa, Özlem Tastan Bishop

**Affiliations:** Research Unit in Bioinformatics (RUBi), Department of Biochemistry and Microbiology, Rhodes University, Makhanda 6140, South Africa; colleen.manyumwa06@gmail.com

**Keywords:** gamma carbonic anhydrase, homology modeling, MD simulations, *betweenness centrality*, MD-TASK, hydrothermal vents, carbon dioxide sequestration

## Abstract

Carbonic anhydrases (CAs) have been identified as ideal catalysts for CO_2_ sequestration. Here, we report the sequence and structural analyses as well as the molecular dynamics (MD) simulations of four γ-CAs from thermophilic bacteria. Three of these, *Persephonella marina*, *Persephonella hydrogeniphila,* and *Thermosulfidibacter takaii* originate from hydrothermal vents and one, *Thermus thermophilus* HB8, from hot springs. Protein sequences were retrieved and aligned with previously characterized γ-CAs, revealing differences in the catalytic pocket residues. Further analysis of the structures following homology modeling revealed a hydrophobic patch in the catalytic pocket, presumed important for CO_2_ binding. Monitoring of proton shuttling residue His69 (*P. marina* γ-CA numbering) during MD simulations of *P. hydrogeniphila* and *P. marina*’s γ-CAs (γ-PhCA and γ-PmCA)*,* showed a different behavior to that observed in the γ-CA of *Escherichia coli,* which periodically coordinates Zn^2+^. This work also involved the search for hotspot residues that contribute to interface stability. Some of these residues were further identified as key in protein communication via *betweenness centrality* metric of dynamic residue network analysis. *T. takaii’s* γ-CA showed marginally lower thermostability compared to the other three γ-CA proteins with an increase in conformations visited at high temperatures being observed. Hydrogen bond analysis revealed important interactions, some unique and others common in all γ-CAs, which contribute to interface formation and thermostability. The seemingly thermostable γ-CA from *T. thermophilus* strangely showed increased unsynchronized residue motions at 423 K. γ-PhCA and γ-PmCA were, however, preliminarily considered suitable as prospective thermostable CO_2_ sequestration agents.

## 1. Introduction

The increasing concentrations of CO_2_ in the atmosphere have resulted in a search for mitigation strategies to counter global warming [1,2]. Biomineralization, a process where CO_2_ is reacted with mineral ions, such as Ca^2+^ or Mg^2+^, to produce mineral carbonates, has been identified as a viable sequestration method [3]. The carbonates produced by this way present a stable source of carbon without the possibility of CO_2_ re-entering the atmosphere. However, a catalyst is normally required for the quick capture of CO_2_. Carbonic anhydrases (CAs) have been identified as preferable catalysts for this reaction, being responsible for the rapid reversible conversion of CO_2_ to HCO_3_^−^ in the presence of water [4]. Eight CA classes have been identified to date, including the α, β, γ, δ, ζ, η, θ, and ι classes, all utilizing a metal ion in the active site, which is predominantly a Zn^2+^ [5,6,7,8,9]. The Zn^2+^ is involved in catalysis by means of a Zn-bound hydroxide molecule, generated by the transfer of a proton from a Zn-bound water. The hydroxide molecule is responsible for the nucleophilic attack on the CO_2_ molecule, producing HCO_3_^−^ [10,11].

Apart from the α- and β-CAs, literature on the γ-CAs has been recently increasing. They have been found in a number of organisms, mostly bacteria, although some have been identified in fungi and plants [12,13,14,15,16]. Those characterized from plants, however, have displayed the inability to catalyze the reversible hydration of CO_2_, but instead, have been observed as essential subunits of mitochondrial complex 1, an enzyme complex in the plant respiratory chain [17,18,19]. All γ-CAs are structurally similar, adopting a homo-trimeric architecture dominated by β-sheets and an α-helical C-terminal. These CAs are only functional in their trimeric form because each active site is located between two monomers, with two coordinating residues coming from one monomer and the third from the neighboring one. The catalytic metal ion in the active site is usually Fe^2+^ but γ-CAs have also been observed to utilize Zn^2+^ and Co^2+^ [12,20,21]. Similar to the α-CAs, γ-CA’s catalytic Zn^2+^ is tetrahedrally coordinated by three His residues and the fourth coordination is fulfilled by a water molecule. Despite the functionality differences of identified γ-CAs, the Zn^2+^ coordination residues have been completely conserved to date [12,15,17]. Variability of residues in the catalytic pocket, however, has been evident in this class. Catalytically active γ-CAs include those from *Esherischia coli* (EcoCA-γ) [22], *Methanosarcina thermophila* (Cam) [13,23], the pathogenic *Porphyromonas gingivalis* (Pg-iCA) [24], *Pseudomonas aeruginosa* PAO1 (PA5540) [14], the cyanobacteria *Nostoc* sp. PCC 7120 [25] and *Thermosynechococcus elongatus* BP-1 (CcmM) [26]. Organisms with γ-CAs whose activity could not be detected include the small plant, *Arabidopsis thaliana* [15], the cyanobacterium *Synechococcus* PCC 7942 [27] as well as *Thermus thermophilus* HB8 [28].

Thermostability is a desirable characteristic in catalysts for biotechnology purposes. This is especially applicable for carbon sequestration at industrial sites, where there is mass production of industrial flue gas at high temperatures, containing large concentrations of CO_2_. Accordingly, the main focus of this study was to assess selected γ-CA proteins in silico for thermostability properties. The γ-CAs were from three organisms, *Persephonella hydrogeniphila* [29], *Persephonella marina* [30] and *Thermosulfidibacter takaii* [31], which were isolated from hydrothermal vent systems. A fourth γ-CA protein, from a hot spring thermophile *Thermus thermophilus* HB8 (γ-TtCA), was included in this study, based on its alleged thermostability properties, for comparison purposes [28].

An in silico approach was motivated by a similar study of the α-CAs including those from *P. hydrogeniphila* and *P. marina*, revealing important functional characteristics of the CAs as well as identifying possible thermostable sequestration agents [32]. Computational studies of the well-studied α-CA from *Thermovibrio ammonificans* have also been performed, where possible residue mutations for enhanced thermostability were identified [33]. The variants expressed after mutation studies indeed produced a more stable CA with an increased tolerance for high temperatures [34]. The prospects for in silico studies are therefore considered monumental and worth exploring.

Here, we retrieved the γ-CA sequences and aligned with previously identified γ-CA sequences for comparison. This included the γ-CA from the hydrothermal vent originating hyperthemophilic archaeon, *Pyrococcus horikoshii* (Cap), whose activity has not yet been determined [35]. The structures for *P. hydrogeniphila, P. marina* and *T. takaii* CAs (γ-PhCA, γ-PmCA and γ-TtkCA respectively) were modeled as trimers substantiating the importance of the interface analysis to identify important interface residues. *T. thermophilus’* crystal structure was already available. Hotspot residues, defined as those whose mutation to alanine results in destabilization of the interface due to their significant energy contribution, were also identified [36]. Analysis of the active site revealed a hydrophobic region proposed to be functional for CO_2_ binding. Molecular dynamics (MD) simulation analyses revealed the conformational behavior of the protein structures at high temperatures. Average betweenness centrality (BC) analysis [37] revealed important residues in protein communication linked to the function of the γ-CAs. Most of these were found in the interface, including some catalytic site residues, also located in the interface. Generally, γ-PhCA and γ-PmCA showed indications of thermostability, including structure rigidity, at high temperatures. This study also unveiled the structural factors behind the lack of catalytic activity previously observed in γ-TtCA [28]. Altogether, this study builds on research on the γ-CAs and simultaneously discloses potential thermostable CO_2_ sequestration agents.

## 2. Results and Discussion

### 2.1. Sequence and Structural Analyses

Residue numbering henceforth is in compliance with γ-PmCA unless explicitly stated. Given that there are three different interfaces, the chain identities of the residues involved will be stated as superscripts in cases where a bond is being discussed.

#### 2.1.1. Multiple Sequence Alignment (MSA)

**Figure 1:**
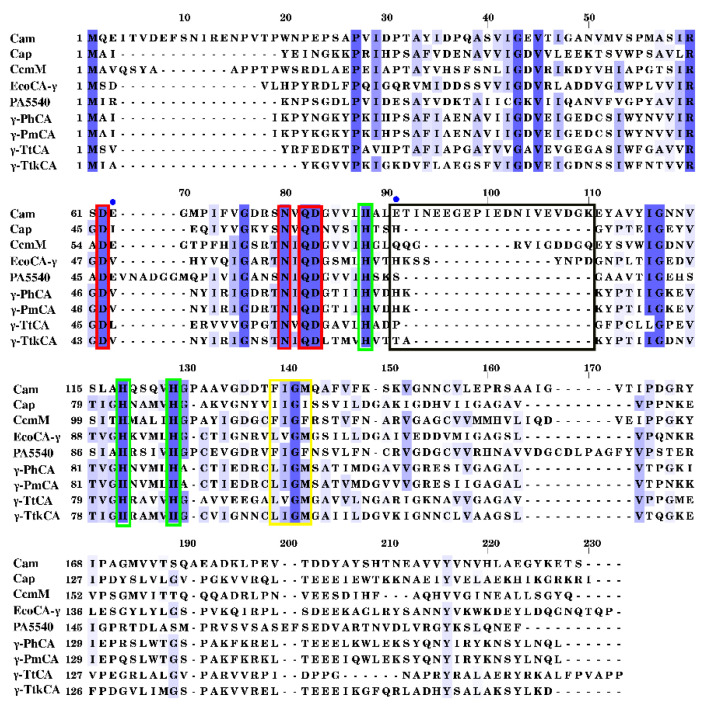
Multiple sequence alignment of the γ-CA sequences performed by PROMALS3D alignment program. Abbreviations: *Methanosarcina thermophile*—Cam; *Pyrococcus horikoshii*—Cap; *Thermosynechococcus elongatus* BP-1—CcmM; *Esherischia coli*—EcoCA-γ; *Pseudomonas aeruginosa*—PA5540; *Persephonella hydrogeniphila*—γ-PhCA; *Persephonella marina*—γ-PmCA; *Thermus thermophilus*—γ-TtCA; and *Thermosulfidibacter takaii*—γ-TtkCA. Residues are colored by the extent of conservation. Residues in the red boxes are important for catalysis while Zn^2+^ coordinating His residues are shown in green boxes. The black box depicts the insert present for Cam, CcmM, and EcoCA-γ. The blue dots show proton shuttling Glu residues identified in Cam. Yellow box depicts residues around the hydrophobic region in the catalytic site of γ-PmCA.

MSA of the γ-CA sequences was performed by PROMALS3D [38] alignment program (Figure 1). *Methanosarcina thermophila* (Cam), *Pyrococcus horikoshii* (Cap), *Thermosynechococcus elongatus* BP-1 (CcmM), *Esherischia coli* (EcoCA-γ), *Pseudomonas aeruginosa* (PA5540), *Persephonella hydrogeniphila* (γ-PhCA), *Persephonella marina* (γ-PmCA), *Thermus thermophilus* (γ-TtCA) and *Thermosulfidibacter takaii* (γ-TtkCA) sequences were included in the MSA. High variability was observed amongst the sequences. Residues that coordinate the catalytic Zn^2+^ metal, His66, His84, His89, were completely conserved across sequences as expected. Additionally, conserved were Arg45, Asp47, Gln60 and Asp61 which play a role in catalysis [28,35,39]. Arg45^ABC^ and Asp61^BCA^ have been reported to form an ion pair and are marked by red boxes in the γ-CA MSA, along with Asp47 and Gln60 [39,40]. The most distinct variation was the insert found in Cam between Leu83 and Gly102 (Cam numbering). Loops for CcmM and EcoCA-γ were also slightly longer than the four CAs being investigated. Two proton transfer residues crucial in the catalytic mechanism for Cam, Glu62 and Glu84 with the former found on the elongated loop, were absent in γ-PhCA, γ-PmCA, γ-TtCA, and γ-TtkCA. In the position for Glu84 (Cam numbering), γ-PhCA and γ-PmCA, similar to Cap and EcoCA-γ, contained a His residue, which has been proposed to be involved in proton transfer [41]. This residue has, however, been observed occasionally coordinating Zn^2+^ in EcoCA-γ, resulting in a “closed” conformation. This in turn, resulted in a mechanism similar to the β-CAs in which they switch between “open” and “closed” states [42,43]. This phenomenon was, thus, searched for γ-PhCA and γ-PmCA following MD simulations. γ-TtCA and γ-TtkCA were different from these CAs, containing a Pro and Thr residue respectively, in this position.

#### 2.1.2. Homology Modeling and Structural Analyses

3D structures of γ-PhCA, γ-PmCA, and γ-TtkCA proteins were modeled as generic γ-CAs, which are known to exist as trimers and all three passed the verification process (Table 1). Verify3D [44] scores were above the threshold of 80% and PROCHECK [45] confirmed no residues to be in the disallowed region thus the model qualities were satisfactory. The crystal structure for γ-TtCA (PDB ID: 6IVE) was used for all further analyses. 

**Figure 2:**
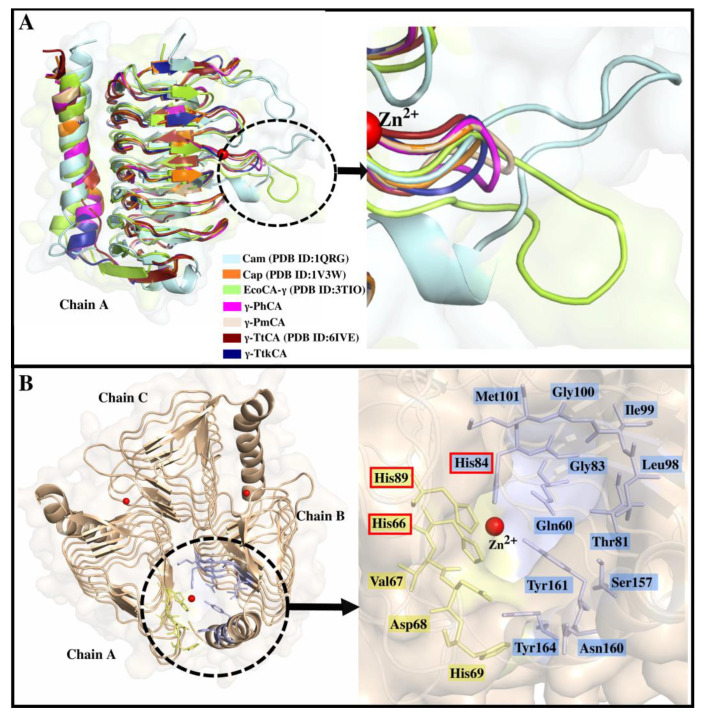
(**A**): Aligned monomeric γ-CA structures of Cam (cyan), Cap (orange), EcoCA-γ (green), γ-PhCA (magenta), γ-PmCA (wheat), γ-TtCA (maroon), and γ-TtkCA (dark blue). The loop region indicated in the MSA is shown by the black dotted circle and zoomed into in the image pointed to by the black arrow. (**B**): Trimeric structure of γ-PmCA with the active site which is located between Chains A and B delimited by the black dotted circle. The black arrow points to the enlarged active site showing the residues that contribute to its formation. Zn^2+^ is shown as a red sphere and the three His residues coordinating it are boxed in red.

Each monomer contained the typical left-handed β-helix resembling a prism-like shape, with loops between β-sheets. The C-terminal was made of a long α-helix. The loop between β-sheet 5 and 6, shown in Figure 2A displayed variability amongst γ-CAs upon comparison with Cam, Cap and EcoCA-γ. γ-PhCA, γ-PmCA, and γ-TtkCA revealed a shorter loop compared to Cam and EcoCA-γ but similar to Cap and γ-TtCA. This region corresponds to the inserts displayed in the MSA (Figure 1).

The open active site of the structures expectedly resembled that of previously crystallized γ-CAs, containing three His residues and a free fourth coordination position to be fulfilled by a water molecule (Figure 2B). This coordination was shared between two monomers. The catalytic pocket was further scrutinized in this study, and other residues were identified as contributing to its surface. Hydrophobic residues Leu98, Ile99, Gly100, and Met101 form a hydrophobic patch at one end of the pocket. This is surmised to play a role in CO_2_ binding similar to the hydrophobic pocket in α-CAs [11,32,46,47]. Met135 (Cam numbering) has been previously noted as part of a possible hydrophobic CO_2_ binding site in Cam, but with a different set of non-conserved residues, including some in the extended loop shown in Figure 2A [23]. It was interesting to observe that the hydrophobicity of this region in the alignment was conserved amongst γ-PhCA, γ-PmCA, and γ-TtkCA as well as previously crystallized Cam, Cap, EcoCA-γ, and γ-TtCA (see yellow box in Figure 1). In the absence of the two Glu proton transfer residues from Cam, a different channel is evidently followed by these CAs, given the high catalytic activity displayed by recently characterized EcoCA-γ. Orientation of Tyr161 towards Zn^2+^ in the catalytic site evinces its role in proton transfer as previously suggested for the γ-CA from *Arabidopsis thaliana* as well as Cap [15,35]. It occupies the structural position of Cam’s Asn202, whose involvement in catalysis has been confirmed. Tyr164, which is also contributing to the catalytic pocket surface (Figure 2B), is also suggested to participate in proton transfer but was absent in both γ-TtCA and γ-TtkCA. It should be pointed out that γ-TtCA did not contain any effective substitutions for proton transfer residues His69 and Tyr164 (γ-PmCA numbering). Both the residues were hydrophobic, disrupting the proton transfer pathway, thus explaining the absence of activity previously observed in vitro [28]. However, the substitute residues for Cam’s Glu62 and γ-PmCA’sTyr164 in γ-TtCA (Leu47^ABC^ and Leu156^BCA^ respectively) were observed to form hydrophobic interactions in the interfaces.

Besides this interaction between monomers, other interactions have been observed following submission of γ-CA structures to the five web servers used to identify interface residues in this study and four of these servers for hotspot residue identification. Importance of the hotspot residues in interface stability is significantly more compared to other interface residues. Results from each server are listed in Supplementary material, Table S1. A consensus of the residues contributing to the interfaces of the structure, consequently supporting the trimeric form of the CAs, is displayed in Table 2. The previously discussed salt bridge formed by Arg45^ABC^ and Asp61^BCA^ was recognized between monomers in all structures. Both these residues were recognized has hotspot residues in all four γ-CAs. Arg45 was also involved in an intra-subunit ionic interaction with Asp47. This chain of ionic bonds is hereby referred to as the Asp-Arg-Asp* ionic triad, with the asterisk indicating a residue from a different monomer. Met101 which is part of the hydrophobic patch in the catalytic pocket was also recognized as a hotspot residue in all γ-CAs. It was interesting to note that γ-TtCA was the only CA amongst the four containing hotspot residues in the N-terminal, specifically Tyr4 and Phe6. Tyr4^ABC^ was observed to form hydrophobic interactions with Leu164^BCA^ and Pro166^BCA^ while Phe6^ABC^ forms hydrophobic interactions with Leu156^BCA^, Tyr160^BCA^, and Leu164^BCA^ in the C-terminal.

Interface characteristics from PDBePISA [48] are detailed in Table S2 (Supplementary material). These revealed the total interface areas, also regarded as the buried surface areas (BSAs) for γ-PhCA and γ-PmCA to be 5155 Å^2^ and 5353 Å^2^ respectively while a lower BSA of 4749 Å^2^ was seen for γ-TtkCA. γ-TtCA showed an extensive BSA of 6846 Å^2^ which, when correlated to the total surface area of the multimer, the BSA accounted for 28% of the protein. For the other three CAs, the BSAs constituted a smaller percentage, approximately 20–21% of the proteins. This correlated with the higher number of hydrogen bonds and salt bridges observed for γ-TtCA using both PDBePISA and Protein Interactions Calculator (PIC) [49] servers compared to the other structures. Occupancy of the hydrogen bonds was further analyzed across MD simulations in a later section (Section 2.2.3). Structures of previously crystallized Cap (PDB ID: 1V3W) and Cam (PDB ID: 1QRM) were also submitted to the PDBePISA web server and were observed to have BSAs of totals 7842 Å^2^ and 6180 Å^2^ respectively, which is irreconcilable to values previously reported (18,775 Å^2^ and 23,352 Å^2^ respectively) [35]. These covered approximately 26.5% and 25% of the total surface area of the structures. Models were thus less closely packed compared to their template as well as Cam.

### 2.2. Molecular Dynamics Simulations

Simulations at increasing temperatures were mainly to identify possible thermostability in the γ-CAs from *P. hydrogeniphila*, *P. marina*, *T. takaii*, and *T. thermophilus*. This was done mostly through conformational dynamics analyses. However, probing protein functionality at residue level gave rise to insights on residues important for stability and function.

#### 2.2.1. Conformational Analysis

**Figure 3:**
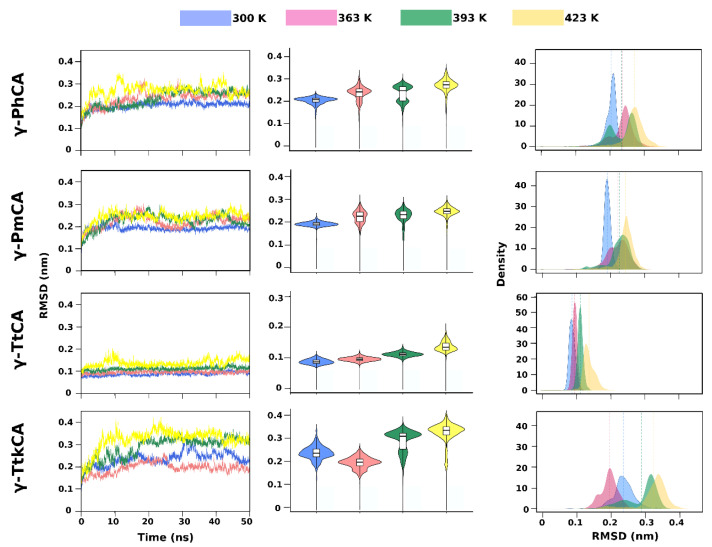
Root mean square deviation (RMSD) line graphs (**left**), violin plots (**middle**), and kernel density estimation (KDE) plots (**right**) of γ-PhCA, γ-PmCA, γ-TtCA and γ-TtkCA at 300 K, 363 K, 393 K and 423 K. Median values in the violin plots are shown by the black line in the white box plots and those in the KDE plots are shown as a line with a color corresponding to the respective plot color.

**Figure 4:**
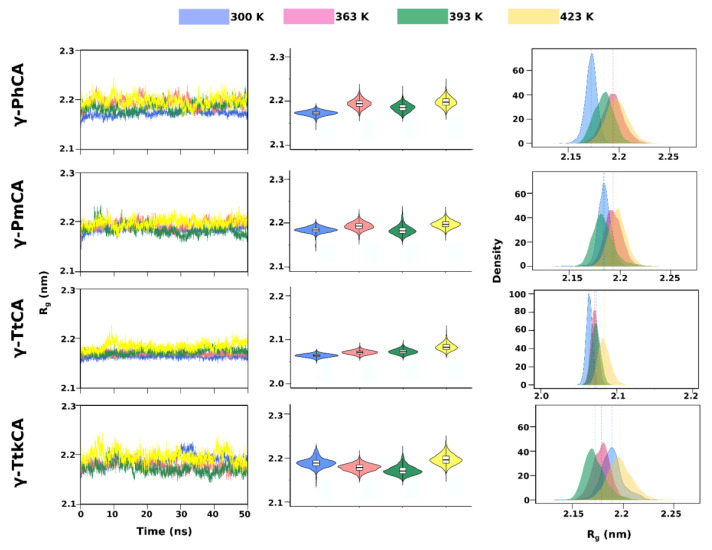
Radius of gyration (R_g_) line graphs (**left**), violin plots (**middle**), and kernel density estimation (KDE) plots (**right**) of γ-PhCA, γ-PmCA, γ-TtCA and γ-TtkCA at 300 K, 363 K, 393 K and 423 K. Median values in the violin plots are shown by the black line in the white box plots and those in the KDE plots are shown as a line with a color corresponding to the respective plot color.

Investigation of the conformations visited by the γ-CAs during simulations at 300 K, 363 K, 393 K, and 423 K was achieved by root mean square deviation (RMSD) and radius of gyration (R_g_) calculations. These were visualized as line graphs, violin plots and kernel density estimation (KDE) plots in Figure 3 (RMSD) and Figure 4 (R_g_). The line graphs convey the evolution of conformations as a function of time, facilitating a perception of particular occurrences such as equilibration and increases or decreases in RMSD/R_g_. The violin plots and the KDE plots illustrate the probability densities of the conformations sampled, enabling the observation of RMSD/R_g_ distribution of conformations across the simulations. Although the violin plots are basically mirrored KDE plots, they also contain a box plots showing the median and interquartile range of the conformational densities.

The RMSD line graphs showed that most structures had attained equilibration by 20 ns. This excluded γ-TtkCA at 300 K and 363 K, as well as γ-PhCA at 393 K. The latter sustained an RMSD between 0.17 nm and 0.2 nm at 393 K, from approximately 3 ns until around 22 ns where a gradual increase was seen before equilibration which was attained around 25 ns. This was conveyed by the KDE and violin plots as two RMSD peaks at 393 K, representing two distinct conformations visited during the simulation. γ-PhCA and γ-PmCA showed an increase in conformations visited at 363 K, indicated by the lower RMSD peaks in the KDE plots. All three different RMSD plots for γ-TtCA show that its structure deviations were lower than those for the other three CAs, with values below 0.15 nm being observed at 300–393 K and a slight increase at 423 K.

In the R_g_ line graphs (Figure 4), all four structures appear to maintain their compactness showing obscure differences across temperatures. The KDE and violin plots displayed a clearer illustration of the conformational occurrences. Some structures at higher temperatures proved to be less compact than those of lower temperature simulations. This observation was seen in the KDE and violin plots of (i) γ-PhCA at 393 K compared to 363 K, (ii) γ-PmCA at 393 K compared to 300 K and 363 K, (iii) γ-TtkCA at 363 K compared to 300 K and (iv) γ-TtkCA at 393 K compared to both 300 K and 363 K. Minimal differences in R_g_ were seen for γ-TtCA, with a notable increase at 423 K, clearly shown by a higher positioned violin plot (yellow) and KDE plot slightly shifted to the right. γ-TtkCA behaved slightly differently from the other three γ-CAs, with a higher number of conformations being visited at 300 K. The density peaks in the KDE plots for γ-TtCA remained more or less the same height with increase in temperature as did those for γ-PhCA and γ-PmCA for the last three temperatures.

It was interesting to note that the increase in RMSD noted in γ-TtkCA at 393 K (Figure 3) corresponded with the structure becoming more compact (decrease in R_g_) whereas the increase in its RMSD at 423 K corresponded with the structure becoming less compact (increase in R_g_).

**Figure 5:**
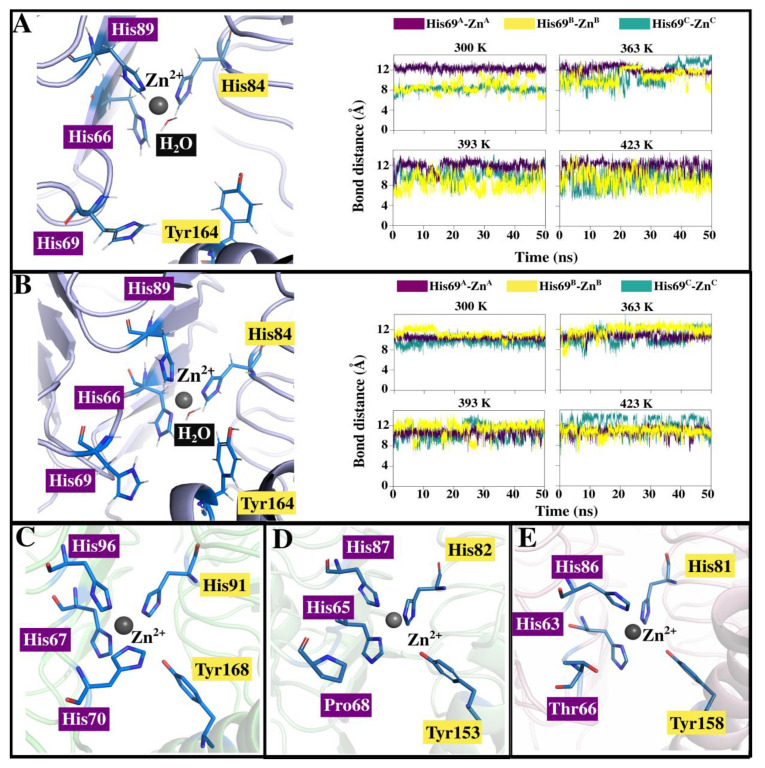
(**A**,**B**) show position of His69 in the active sites of γ-PhCA and γ-PmCA respectively as well as plots illustrating its bond distance from Zn^2+^ during simulations at 300 K, 363 K, 393 K and 423 K. (**C**) shows His70 taking up the fourth coordination position in EcoCA-γ. (**D**,**E**) show the active site of γ-TtCA and γ-TtkCA, with Pro68 and Thr66 respectively, in place of γ-PhCA’s His69.

Further, the distance between δ-nitrogen atom (ND1) of His69 and Zn^2+^ in catalytic sites for γ-PhCA and γ-PmCA (see structures in Figure 5A,B) was monitored throughout all trajectories to see if a possible “closed” conformation as the one observed in EcoCA-γ (Figure 5C) was being exhibited. The Zn-His coordination bond distance is 2.09 ± 0.14 Å [50,51]. In all cases, the active site remained open with His69 staying more than 5 Å away from Zn^2+^ (see plots in Figure 5A,B). Coordination of water molecules to Zn^2+^ was also observed during simulations and is shown for γ-PhCA and γ-PmCA structures. Proximity of His69 to the active site as well as a similar spatial orientation as the one in EcoCA-γ corroborates its role in proton transfer [41]. The loop on which EcoCA-γ’s His70 is however longer as observed in Figure 2A compared to γ-PhCA and γ-PmCA, possibly allowing flexibility for Zn^2+^ coordination. This investigation was irrelevant for γ-TtCA and γ-TtkCA on account of the presence of Pro68 and Thr65 respectively in place of the His residue (Figure 5D,E). Unlike the non-polar Pro residue, Thr has been identified in other proton transfer pathways [52], due to its capability to donate and accept hydrogen atoms, thus this substitution in γ-TtkCA might have little to no effect on proton shuttling in this CA.

#### 2.2.2. RMSF and Average Betweenness Centrality (BC) Analyses

**Figure 6:**
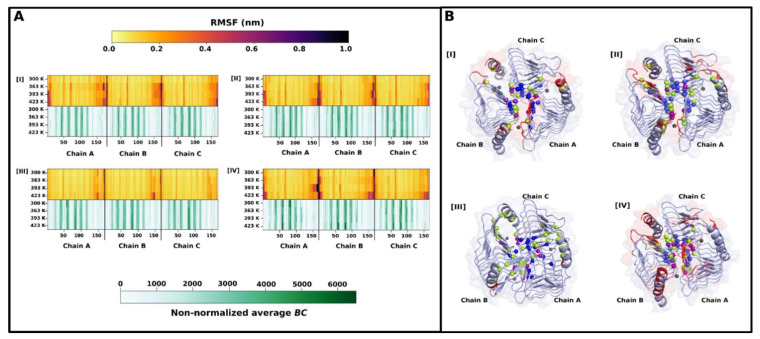
(**A**): Heat maps of root mean square fluctuation (RMSF) (**top**) and average *BC* (**bottom**) of residues from I—γ-PhCA, II—γ-PmCA, III—γ-TtCA and IV- γ-TtkCA. (**B**): Structures of I—γ-PhCA, II—γ-PmCA, III—γ-TtCA and IV—γ-TtkCA. Hotspot residues (green), top 5% average *BC* residues (blue) and an intersection of the two (purple) are mapped as spheres. Regions fluctuating above 0.4 nm at 423 K were colored red. Zn^2+^ metal ions are depicted as grey spheres.

Generally, the γ-CA simulations displayed remarkable rigidity with increase in simulation temperatures, shown by root mean square fluctuation (RMSF) heat maps in Figure 6A. This is expected given their intricate folding with numerous beta sheets across the structures. Fluctuation was mainly seen in the beta-sheet and successive loop making up the N-terminal as well as number of residues in the C-terminal helix. This was not the case, however, for γ-TtCA whose N-terminals barely displayed any fluctuation compared to the other three CAs. The N-terminal for each chain is generally oriented close to the C-terminal of the neighboring monomer, and in the case for γ-TtCA, the C-terminal has a unique beta-sheet, extending over the N-terminal (Figure 6B). These regions are close enough for interactions, which are seemingly responsible for the reduced RMSF values. These interactions are further probed in Section 2.2.3. Beta-sheets are known to be rigid and exhibit low fluctuations. It is worth recalling that γ-TtCA had hotspot residues in the N-terminal (Table 2) unlike the rest of the CAs, also illustrated in Figure 6B (III). This observation accounts for γ-TtCA’s larger BSA noted in Section 2.1.2 during interface analysis. *Betweenness centrality* (*BC*) quantifies how involved a residue is in the relay of information within a protein’s residue interaction network. Average *BC* is accordingly the mean of *BC* values calculated for specified number of frames across a trajectory and is used as an indication of residue usage [37]. Regions that were fluctuating considerably coincided with residues exhibiting low average *BC* values, which are regarded as low communication residues. This is in alignment with the inverse relationship between RMSF and *BC* originally observed by Penkler et al. [53]. The top 5% highest communication residues with high average *BC* values, were picked for further analysis. These are outlined in Table 3 and are mapped onto structures of γ-PhCA, γ-PmCA, γ-TtCA and γ-TtkCA in Figure 6B. Location of these residues, along with other identified hotspot residues (Section 2.1.2), was observed towards the center of the trimer. A number of high communication residues coincided with interface and hotspot residues. γ-PhCA and γ-PmCA had hotspot residues toward the end of the C-terminal, specifically, Tyr164 and Tyr168. Tyr164 was previously mentioned as important in the formation of catalytic pocket and possibly in proton transfer. Zn^2+^ coordinating residues were also found appearing in all four proteins. His66 was observed in γ-PmCA, γ-TtCA, and γ-TtkCA, and formed ionic interaction with Asp61 of the Asp-Arg-Asp* ionic triad. CO_2_ binding pocket residue Met101 appeared in one or more chains for all proteins. Met98 for γ-TtkCA formed hydrophobic interactions with interface residue Met84 as well as hotspot residue Ile101, both which were identified as high average *BC* residues in one or more chains. The residue equivalents of γ-TtkCA’s Met84 in γ-PmCA and γ-TtCA are Met87 and Val85 respectively in the MSA, and this was also a high usage residue in both CAs, forming hydrophobic interactions with M99 (γ-TtCA numbering) in the CO_2_ binding pocket. This was not observed in the top 5% residues for γ-PhCA. Asn41 which was an interface residue in γ-PmCA and a hotspot residue in γ-PhCA and γ-TtkCA appeared with high average *BC* values in all three proteins. γ-TtCA had Gly40 in this position, which is also listed in Table 3 as a high communication residue.

#### 2.2.3. Hydrogen Bond Analysis

**Figure 7:**
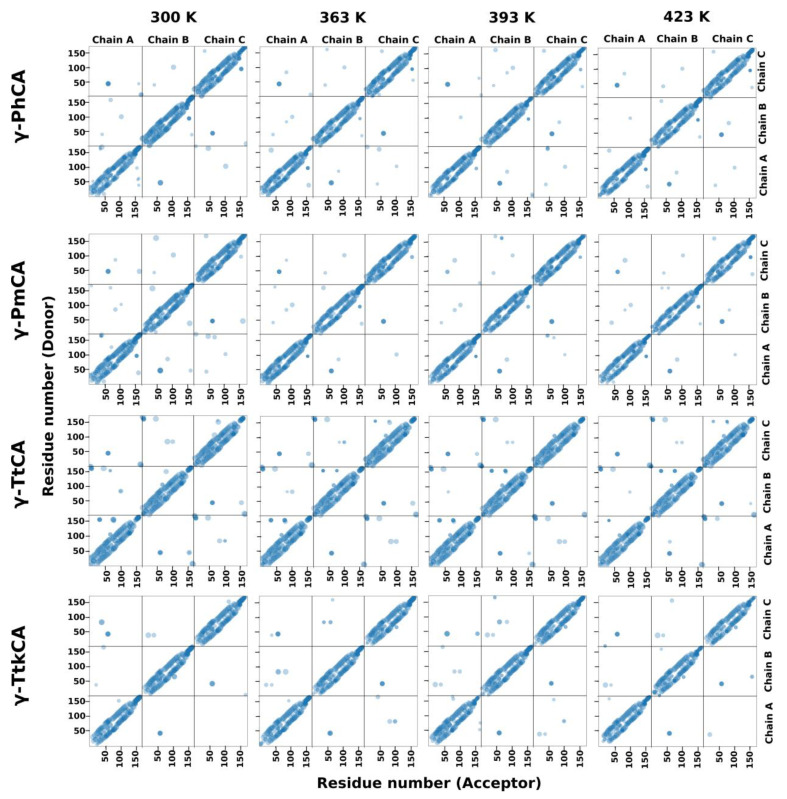
Hydrogen bond plots for γ-PhCA, γ-PmCA, γ-TtCA, and γ-TtkCA at 300 K, 363 K, 393 K, and 423 K. Hydrogen bonds present for ≥25% of the simulation are illustrated as translucent blue spheres and the color intensity increase with an increase in bonds formed by those particular residues. Sphere size is directly proportional to the fraction of the simulation a hydrogen bond was present, i.e., the larger the spheres show the longer the bond was present and vice-versa.

Hydrogen bonds are important interactions in protein structures and their occurrence across the trimers and their evolution with increase in temperatures was studied using hydrogen bond analysis. These are represented as scatterplots in Figure 7. An intricate network of hydrogen bonds was observed within the individual chains for all CAs. Those in the interfaces were significantly less, a phenomenon predicted during interface analysis (Table S1, Supplementary material), and a slight decrease in bonds was observed at higher temperatures. An increase in temperature did not appear to cause a perceivable change in the intra-subunit hydrogen bonds. γ-TtCA had noticeably more hydrogen bonds in the interface compared to the other three CAs and γ-TtkCA had the least. The most prominent hydrogen bonds in all interfaces for all four CAs were those formed between Arg45^ABC^ and Asp61^BCA^ which were previously mentioned to form an ionic bond. Multiple bonds between these two residues were observed across all four temperatures. In γ-TtCA, some of the hydrogen bonds were persistent in all three interfaces at all four temperatures. The terminals were observed to have a number of hydrogen bonds between Val3^ABC^–Val167^BCA^, Arg5^ABC^–Phe165^BCA^, Glu7^ABC^–Arg159^BCA^ and Glu7^ABC^–Ala163^BCA^, which supports the RMSF results showing low fluctuations in the terminals. Another such bond was seen between hotspot residue Tyr160^ACB^ and Asp46^CBA^, of which the latter was only observed as a hotspot residue in chain C. In light of the sparsity of hydrogen bonds in the interface regions, the trimers are considered to be stabilized mostly by hydrophobic interactions, most of which have been identified during interface analysis as well as average *BC* analysis.

#### 2.2.4. Dynamic Cross Correlation Analysis

**Figure 8:**
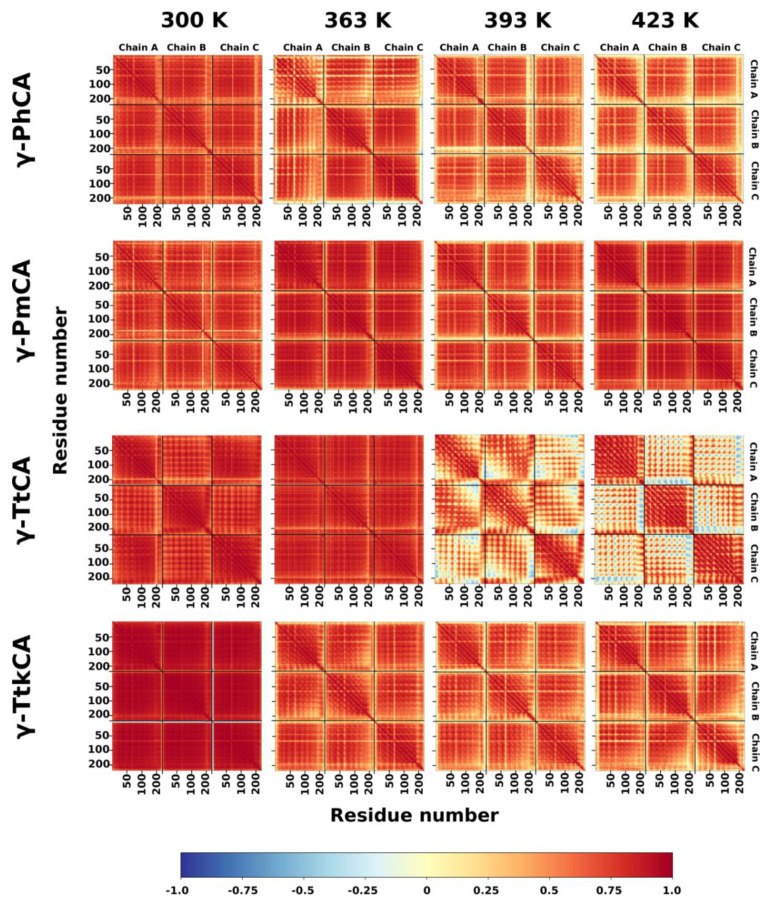
Dynamic cross correlation (DCC) heat maps for γ-PhCA, γ-PmCA, γ-TtCA, and γ-TtkCA at 300 K, 363 K, 393 K, and 423 K.

Dynamic cross correlation (DCC) was utilized to observe the extent of concerted movements in the same direction across the simulations and how these are affected by temperature. Residue behavior differed from protein to protein as shown in Figure 8. γ-PhCA and γ-TtkCA displayed a decrease in correlated motions at 363 K, mostly in the terminal regions and maintained nearly the same correlations at 393 K and 423 K. γ-PmCA showed the high correlated residue motions at all four temperatures, with the highest being observed at 363 K and 423 K. The behavior for γ-TtCA residues was quite peculiar, manifesting some anti-correlated motions between chains at 393 K which became even more pronounced at 423 K, especially for residue pairs involving C-terminal residues. A distinct increase in RMSF of residues around this region was correspondingly observed in Figure 6A (III) at 423 K. Given that the catalytic sites are located between chains, it was important that movements of the constituent residues were correlated. Further investigation revealed positive correlations between each active site’s residues at both 393 K and 423 K.

## 3. Materials and Methods

**Figure 9:**
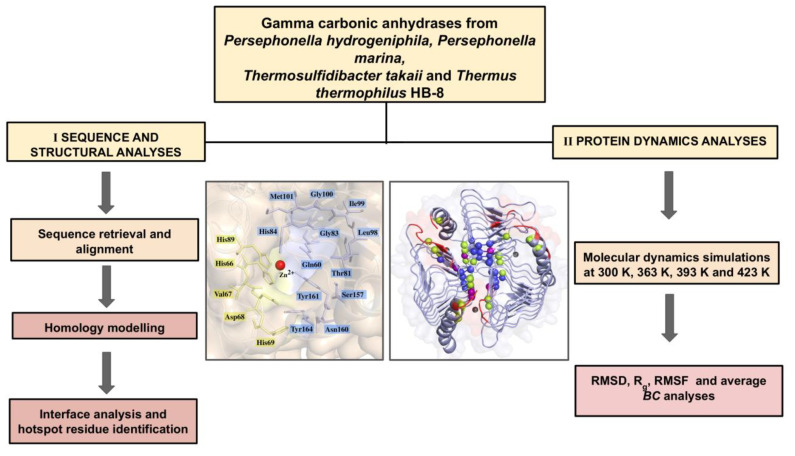
General methodology followed in this work. RMSD, R_g_, RMSF, and *BC* refer to root mean square deviation, radius of gyration, root mean square fluctuation and *betweenness centrality* respectively.

The present work comprised of sequence retrieval of four γ-CAs followed by static and dynamic structural analyses of these proteins. The workflow is illustrated in Figure 9.

### 3.1. Sequence Retrieval and Alignment

The thermophiles *Persephonella hydrogeniphila* [29], *Persephonella marina* [30] *Thermosulfidibacter takaii* [31], and *Thermus thermophilus* HB8 [28] were identified through literature search. The α-CA proteins from *P. hydrogeniphila* and *P. marina* have been previously investigated [32,54,55]. γ-CA sequences for these organisms were queried and acquired from the National Center for Biotechnology Information (NCBI) database [56] and have accession numbers WP_097000475 (*P. hydrogeniphila*, γ-PhCA), WP_012675364 (*P. marina*, γ-PmCA) and WP_083498668 (*T. takaii*, γ-TtkCA). The sequence for *T. thermophilus’* CA (γ-TtCA) was obtained from the Protein Data Bank (PDB) [57] with the PDB ID 6IVE. Sequences of previously characterized γ-CAs were also queried from the NCBI as well as the PDB and included for the multiple sequence alignment (MSA). These include γ-CAs from *Escherichia coli* (UniProtKB Accession number: P0A9W9.1, EcoCA-γ) [22], *Methanosarcina thermophila* (PDB ID:1QRL, Cam) [13,23], *Pseudomonas aeruginosa* (GenBank Reference number: QLJ92275.1, PA5540) [14], *Pyrococcus horikoshii* (PDB ID: 1V3W, Cap) [35] *Thermosynechococcus elongatus* BP-1 (PDB ID:3KWD, CcmM) [26]. PROfile Multiple Alignment with predicted Local Structures and 3D constraints (PROMALS3D) [38] was utilized to execute the MSA and to include structural information for the crystallized CAs, their respective PDB IDs and chain identities were provided. Visualization was performed using Jalview v2 [58].

### 3.2. Homology Modeling

Homology models for γ-PhCA, γ-PmCA, and γ-TtkCA were calculated using the γ-CA structure from the hyperthermophilic archaeon, *P. horikoshii* (Cap, PDB ID: 1V3W) [35]. It was identified as the most suitable template for all three γ-CA sequences, with template-sequence identities and coverages above 43% and 97% respectively. For each of the sequences, the slow refinement option and the automodel class in MODELLER version 9.20 [59] were used to generate 100 trimeric models, including the Zn^2+^ HETATM in the active site. Initial validation of the models was achieved using z-DOPE score calculations, with the top five showing the lowest scores proceeding for evaluation using PROCHECK [45] and Verify3D [44] web servers. γ-TtCA’s structure (PDB ID: 6IVE) was obtained from the PDB database and used going forward.

### 3.3. Interface Analysis and Hot Spot Identification

The Protein Interactions Calculator (PIC) web server [49] was used to survey hydrophobic and ionic interactions across the trimers. Hotregion [60], HSPred [61], PPCheck [62], Protein Interfaces, Surfaces and Assemblies (PDBePISA) [48] and Robetta [36] web servers were queried for interface residues. These web servers except PDBePISA were also searched for hotspot residues. In both instances, interface and hotspot residues concurring in at least three servers were considered in this study. PyMOL v1.7.2.1 [63] was used to map these residues onto the models.

### 3.4. Molecular Dynamics Simulations 

Carbonation of metals during sequestration is favored by alkaline pHs. Consequently, γ-PhCA, γ-PmCA, γ-TtCA, and γ-TtkCA were protonated at pH 8 using H++ web server [64]. Given the Zn^2+^ coordinating residues for γ-CAs are the same as in α-CAs, previously generated and validated parameters for α-CAs were inferred onto these structures prior to simulation [32,65]. Correctness of protonation states of the His residues was verified manually by checking the PDB files. His66 (γ-PmCA numbering) (HIE) coordinates the Zn^2+^ by the δ-nitrogen with the ε-nitrogen protonated, and the other two His (HID) residues are the reverse. *tleap* [66] from the AmberTools20 [67] package was used alongside ACPYPE [68] to generate coordinate and topology files utilizing the AMBER ff14SB force field [69]. A TIP3P cubic water box with a clearance space of 10 Å was employed in the solvation of the systems. Steepest descent minimization of the systems was performed using GROMACS v2016.1 [70], and this process was complete upon attainment of a maximum force < 1000 kj mol^−1^ nm^−1^. Canonical ensemble equilibration followed by isothermal-isobaric equilibration were performed for 100 ps each at four separate temperatures of 300 K, 363 K, 393 K and 423 K. MD simulations of 50 ns proceeded at these same temperatures for each γ-CA. These calculations as well as trajectory analyses for root mean square deviation (RMSD), radius of gyration (R_g_) and root mean square fluctuation (RMSF) were also performed using GROMACS v2016.1. Simulations were run using a total of 12,806 CPU hours on the Center for High Performance Computing (CHPC) Cape Town, South Africa. Distance between His69 (γ-PmCA numbering) and Zn^2+^ in each active site was monitored using Visual Molecular Dynamics (VMD) [71] and plotted in Gnuplot 5.2 [72]. A maximum bond distance of 3.5 Å was applied for hydrogen bond analysis of the trimers which was carried out using AmberTools20′s *cpptraj* [73].

### 3.5. Average Betweenness Centrality Analysis

The frequency of residue usage during MD simulations was calculated using *betweenness centrality* (*BC*) analysis in MD-TASK (Research Unit in Bioinformatics, Makhanda, South Africa) [37]. Dynamic residue networks are constructed using C_α_ atoms (C_β_ for Gly), regarded as nodes and an edge is created when two nodes are within a certain distance from each other. In this study the maximum distance between two nodes was specified as 6.7 Å. Using the *calc_network.py* script, the shortest paths between two nodes in the residue interaction network were calculated for each 100th frame over the last 25 ns of each trajectory. In every frame used, *BC* was calculated for each residue as the total number of shortest paths going through it using the calc-BC option in the *calc_network.py* script. The *avg_network.py* script was then utilized to calculate the average *BC* for each residue from those calculated for each used frame by the *calc_network.py* script.

### 3.6. Dynamic Cross Correlation Analysis

In order to analyze the pairwise residue correlated motions in each trimer, the displacement of C_α_ atoms (C_β_ for Gly), from their initial position was calculated using the *calc_correlation.py* script in MD-TASK [33]. A time step of 100 ps was specified and dynamic cross correlation (DCC) was calculated for the full 50 ns trajectories. Python scripts were used to plot heat maps from the correlation matrices produced.

## 4. Conclusions

In order to investigate the potential use of γ-CAs as CO_2_ sequestration agents as well as to achieve an understanding of the functional properties of these proteins, we applied a number of computational techniques in the analysis of four γ-CAs from bacteria found in high temperature environments. These bacteria included *P. hydrogeniphila*, *P. marina*, *T. takaii* and *T. thermophilus*. Alignment of these sequences with those of other known γ-CAs revealed low levels of conservation within the class. Variability was the most noticeable for proton transfer residues, with their absence in γ-TtCA evincing its lack of activity. Active site similarities between EcoCA-γ and the two *Persephonella* γ-CA proteins, particularly the His residue which is involved in proton transfer, suggest that the latter two might be as active as the former γ-CA which has been recently characterized. This His residue was also present for the γ-CA from *P. horikoshii* which was also isolated from a hydrothermal vent. Dissimilar to EcoCA-γ, however, this residue was not perceived coordinating the catalytic Zn^2+^ during simulations in γ-PhCA and γ-PmCA. The occurrence of a “closed” active site in γ-CAs is thus proposed to be hinged on two features, the first being the presence of a residue capable of coordinating Zn^2+^ residing on the loop after the first Zn^2+^ coordinating His (His66 γ-PmCA numbering) and the second feature being that the loop is elongated to allow flexibility of this particular residue to bond with Zn^2+^. This projection thus raises the question if Cam also periodically exhibits a “closed” active site, since the proton transfer residue, Glu is capable of coordinating Zn^2+^. Future studies could possibly utilize MD simulations to address this, given that the structure was crystallized with an open active site. 

Following the modelling of trimeric structures for γ-PhCA, γ-PmCA, and γ-TtkCA, a hydrophobic region for CO_2_ binding was observed in the catalytic pocket, containing a set of residues whose hydrophobicity was conserved in all sequences in the MSA. This region was clearly defined and illustrated using the structure from γ-PmCA. One of these hydrophobic residues, Met101, was identified as a hotspot residue during interface analysis, which was supported by average *BC* results identifying it as a high usage residue. Other hotspot residues which had high average *BC*s had functional significance, such as the Zn^2+^ coordinating His66, some CO_2_ binding pocket as well as proton transfer residues. This was expected considering the catalytic pocket is shared in the interface of two chains, which was dominated by hydrophobic interactions. Except for the termini regions, RMSF analysis of the simulations revealed the rigidity of the structures at all four temperatures simulated (300 K, 363 K, 393 K and 423 K), particularly in high communication residues, advocating the use of these γ-CAs at high temperatures. Given that γ-TtCA showed exceptional stability properties, the residues identified in this study that are in place of the proton transfer residues, could be mutated in future research to permit and enhance catalytic activity. By doing so, γ-TtCA could be an excellent option for sequestration. This in silico work concludes that γ-CAs from *P. hydrogeniphila* and *P. marina* are viable prospects as CO_2_ sequestration agents. It also adds to biotechnology advances in identification of important features for catalysis and thermostability in the γ-CAs as catalysts for biomineralization of CO_2_.

## Figures and Tables

**Table 1:** Template coverage, sequence identity and model validation for γ-CA sequences.

CA	Organism	Template PDB ID	Template—Sequence Identity	Template—Sequence Coverage	z-DOPE Score	Verify 3D (%)	Procheck	
							Most Favored Region (%)	Disallowed Region (%)
γ-PhCA	*Persephonella hydrogeniphila*	1V3W	46%	98%	−1.42	89.6	86.6	0
γ-PmCA	*Persephonella marina*		46%	98%	−1.62	88.6	88.8	0
γ-TtkCA	*Thermosulfidibacter takaii*		44%	99%	−1.80	89.9	89.9	0

**Table 2:** Residues common to three programs identified as participating in interface formation. Hotspot residues are in bold.

CA	Residues
γ-PhCA	**Interface AB**
	**A:** M1, A2, I4, K5, P6, Y7, N8, V25, I27, N41, V43, **R45**, D47, T63, **I64**, H66, H69, K70, N85, V86, M87, H89, T104, I105, M106, L122, S138
	**B:** E22, N23, W39, **Y40**, N41, Q60, **D61**, G62, **H84**, **N85**, **M101**, S102, A119, T136, W153, N160, Y161, Y164, S167, **Y168**, Q171
	**Interface BC**
	**B:** I4, K5, P6, Y7, N8, V25, I27, **N41**, V43, **R45**, **D47**, V48, G62, T63, **I64**, H66, D68, H69, K70, N85, V86, M87, H89, S102, T104, I105, M106, L122, S138
	**C:** N23, W39, **Y40**, N41, Q60, **D61**, G62, **H84**, **N85**, **M101**, S102, A119, T136, W153, K156, N160, Y161, **Y164**, S167, Y168, Q171
	**Interface AC:**
	**A:** N23, W39, **Y40**, N41, Q60, **D61**, H84, N85, M101, S102, A119, T136, N160, Y161, **Y164**, S167, Y168, N170, Q171
	**C:** A2, I4, K5, Y7, N8, V25, I27, N41, V43, **R45**, D47, G62, T63, **I64**, H66, H69, V86, M87, H89, S102, T104, M106, D107, L122, S138
γ-PmCA	**Interface AB**
	**A:** I3, I4, K5, P6, Y7, K8, Y11, N23, V25, I27, N41, V42, V43, **R45**, D47, V48, I64, **H66**, V67, D68, H69, N85, V86, M87, H89, A102, T104, M106, L122, S138
	**B:** N23, W39, **Y40**, N41, Q60, **D61**, **H84**, N85, **M101**, S102, A119, T136, W153, N160, Y161, **Y164**, S167, **Y168**, N170, Q171, L172
	**Interface BC**
	**B:** I3, I4, K5, P6, Y7, K8, V25, **I27**, N41, V43, **R45**, **D47**, V48, G62, T63, **I64**, H66, H69, N85, V86, M87, H89, S102, T104, V105, M106, L122, S138
	**C:** N23, W39, **Y40**, N41, Q60, **D61**, **H84**, **N85**, **M101**, S102, A119, T136, N160, Y161, Y164, S167, **Y168**, N170, Q171, L172
	**Interface AC:**
	**A:** E22, N23, W39, **Y40**, N41, **D61**, H84, N85, **M101**, S102, A119, G120, W153, N160, Y161, **Y164**, S167, **Y168**, Q171, L172
	**C:** M1, A2, I3, I4, K5, P6, Y7, K8, V25, I27, N41, V43, **R45**, D47, G62, **I64**, H66, D68, H69, **N85**, M87, H89, S102, T104, V105, M106, L122
γ-TtCA	**Interface AB**
	**A:** S2, V3, **Y4**, R5, **F6**, E7, K9, T10, Y24, V26, V42, **R44**, D46, L47, **V63**, **H65**, **R83**, V85, H87, G100, A101, V102, L104, V120, V136
	**B:** W38, **F39**, Q59, **D60**, **H82**, **R83**, **M99**, A117, G118, V136, R152, **Y153**, L156, R159, **Y160**, A163, **L164**, F165, V167
	**Interface BC**
	**B:** V3, **Y4**, R5, **F6**, E7, Y24, **V26**, V42, **R44**, D46, L47, **V63**, **H65**, **R83**, V85, H87, V102, L104, V120, V136
	**C:** P21, G22, Y24, W38, **F39**, Q59, **D60**, **H82**, **R83**, M99, A117, G118, V136, R152, **Y153**, **L156**, R159, **Y160**, A163, **L164**, F165, P166, V167
	**Interface AC:**
	**A:** P21, G22, W38, **F39**, Q59, **D60**, **H82**, **R83**, **M99**, A117, G118, L134, V136, R152, **Y153**, **L156**, R159, **Y160**, A163, L164, F165, P166, V167, A168, T169
	**C:** M1, S2, V3, **Y4**, R5, **F6**, E7, T10, Y24, V26, V42, **R44**, **D46**, L47, **V63**, **H65**, D67, P68, R83, A84, V85, H87, G100, A101, V102, L104, V120, V136
γ-TtkCA	**Interface AB**
	**A:** I2, Y4, K5, **F22**, I24, **N38**, T39, **V40**, **R42**, D44, V45, **L59**, T60, M61, **H63**, R82, A83, M84, H86, **I101**, L103, L119, S135, P136
	**B:** E19, G20, W36, **F37**, N38, Q57, **D58**, **L59**, **H81**, **R82**, M98, G99, A116, G117, M133, H157, Y158, L161, Y165, D168
	**Interface BC**
	**B:** Y4, K5, V7, **F22**, I24, N38, V40, **R42**, D44, V45, L59, T60, **M61**, H63, R82, M84, H86, I101, L103, L119, S135
	**C:** E19, W36, **F37**, **N38**, Q57, **D58**, **L59**, **H81**, **R82**, **M98**, A116, G117, M133, H157, Y158, L161, Y165, D168
	**Interface AC:**
	**A:** E19, G20, W36, **F37**, N38, Q57, **D58**, L59, H81, R82, **M98**, G99, A116, G117, H157, Y158, L161, Y165, D168
	**C:** Y4, K5, G6, F22, I24, N38, V40, **R42**, D44, V45, L59, T60, M61, H63, R82, M84, H86, G99, I101, L103, L119, S135

**Table 3:** Top 5% average *betweenness centrality* (*BC*) residues. Interface residues are in bold text and hotspot residues are bold and italicized.

CA	Residue
γ-PhCA	**Chain A: N41**, V42, ***D61***, **T63**, ***I64***, **V86**, **H89**^h^, **M101 ^c^, A103**, **T104**
	**Chain B: *N41***, V42, T63, ***I64***, ***H84***^h^, **V86**, **A103**
	**Chain C: N41**, V42, ***D61***, T63, ***H84***^h^, **V86**, **H89**^h^, ***M101***^c^, **A103**, **T104**
γ-PmCA	**Chain A: N41**, V42, T63, **I64**, **N85**, **V86**, A103, **A119**
	**Chain B: N41**, V42, **T63**, ***I64***, **H66**^h^, ***H84***^h^, **V86**, ***M101***^c^, A103
	**Chain C: N41**, V42, **V43**, T63, I64, V82, ***H84***^h^, V86, **M87**, **T104**
γ-TtCA	**Chain A:G40**, **N57**, **Q59**, ***D60***, **A62**, ***V63***, ***H65***^h^, ***H82***^h^, **V85**, V86
	**Chain B:** G40, ***D60***, ***H65*** ^h^, ***H82*** ^h^, A84, **V85**, ***M99*** ^c^
	**Chain C:** G40, **V42**, ***D60***, A62, ***H82*** ^h^, **A84**, **V85**, **H87** ^h^, V97 ^c^
γ-TtkCA	**Chain A: *N38***, T39, ***D58***, **T60**, **M61**, ***H63***^h^, **H81**^h^, **R82**, **A83**, **M84**
	**Chain B:** T39, ***D58***, ***L59***, **T60**, ***H81*** ^h^, **A83**, **M84**, **I101**
	**Chain C:** T39, ***L59***, **T60**, **M61**, ***H81*** ^h^, **A83**, **M84**, **M98** ^c^

^h^—Active site His residue; ^c^—CO_2_ binding pocket residue.

## Data Availability

The data generated in the present study are included in this published article. Protein models are available from the corresponding author upon reasonable request.

## Supplementary Materials

Supplementary Materials can be found at https://www.mdpi.com/1422-0067/22/6/2861/s1.

## Abbreviations

*BC*	*betweenness centrality*
CA	Carbonic anhydrase
Cam	Methanosarcina thermophila gamma carbonic anhydrase
Cap	*Pyrococcus horikoshii* gamma carbonic anhydrase
CcmM	*Thermosynechococcus elongatus* BP-1 gamma carbonic anhydrase
EcoCA-γ	*Escherichia coli* gamma carbonic anhydrase
γ-PhCA	*Persephonella hydrogeniphila* gamma carbonic anhydrase
γ-PmCA	*Persephonella marina* gamma carbonic anhydrase
γ-TtkCA	Thermosulfidibacter takaii gamma carbonic anhydrase
MD	Molecular dynamics
MSA	Multiple sequence alignment
PA5540	*Pseudomonas aeruginosa* gamma carbonic anhydrase
R_g_	Radius of gyration
RMSD	Root mean square deviation
RMSF	Root mean square fluctuation
