# Supplementary material for: In Silico Investigation of Potential Applications of Gamma Carbonic Anhydrases as Catalysts of CO2 Biomineralization Processes: A Visit to the Thermophilic Bacteria Persephonella hydrogeniphila, Persephonella marina, Thermosulfidibacter takaii, and Thermus thermophilus
Source: Int J Mol Sci. 2021 Mar 11;22(6):2861. doi: 10.3390/ijms22062861 (PMC8000050; doi:10.3390/ijms22062861)
Supplement: Supplementary file 1 [file ijms-22-02861-s001.pdf]

Supplementary data: *In silico* investigation of potential applications of gamma carbonic anhydrases as catalysts of CO2 biomineralization processes: A visit to the thermophilic bacteria *Persephonella hydrogeniphila, Persephonella marina, Thermosulfidibacter takaii* and *Thermus thermophilus*

Colleen Varaidzo Manyumwa[1] **and Özlem Tastan Bishop**[1]*

[1] Research Unit in Bioinformatics (RUBi), Department of Biochemistry and Microbiology, Rhodes University, Makhanda, 6140, South Africa; colleen.manyumwa06@gmail.com; O.TastanBishop@ru.ac.za

*Correspondence: O.TastanBishop@ru.ac.za; Tel: +27-46-603-8072; Fax: +27-46-603-7576

Table S1: Interface residues for each γ-CAs predicted by the five web servers. Hotspot residues are highlighted in bold.

Carbonic anhydrase		PPCheck	HSPred	Hotregion	PDBePISA	Robetta
γ-PhCA	**Interface AB**	**CHAIN A:** M1, A2, I3, I4, K5, P6, Y7, N8, G9, K10, E22, N23, A24, V25, I26, I27, G28, D29, Y40, **N41**, V42, V43, I44, **R45**, G46, D47, V48, D61, G62, T63, **I64**, I65, H66, V67, D68, H69, K70, H84, N85, V86, M87, L88, H89, C91, M101, S102, A103, T104, I105, M106, D107, G120, A121, L122, V123, T124, P125, S138, P139<br>**CHAIN B:** F19, A21, E22, N23, A24, V25, S37, I38, W39, **Y40**, **N41**, V42, V43, N58, I59, Q60, **D61**, G62, T63, I64, T81, V82, G83, H84, **N85**, V86, L98, G100, **M101**, S102, A103, V117, G118, A119, G120, A121, T136, G137, S138, K143, W153, K156, N160, Y161, R163, Y164, K165, S167, **Y168**, L169, N170, Q171	**CHAIN A:** M1, I4, K5, Y7, N8, V25, I27, N41, **V43**, **R45**, **D47**, **I64**, **H66**, H69, K70, N85, V86, M87, H89, S102, T104, I105, M106, L122, S138,<br>**CHAIN B:** E22, N23, **W39**, **Y40**, N41, **Q60**, **D61**, **H84**, N85, M101, S102, W153, N160, Y161, **Y164**, S167, Y168, Q171	**CHAIN A:** M1, I4, P6, Y7, N8, **V25, I27**, N41, **V43**, **R45**, D47, **I64**, **H66**, H69, N85, **M87**, **H89**, **T104, I105, M106**, L122,<br>**CHAIN B:** E22, N23, **W39**, **Y40**, N41, **D61**, **H84**, N85, **M101**, S102, **A119**, N160, **Y161**, **Y164**, **Y168,** Q171	**CHAIN A:** M1, A2, I4, K5, P6, Y7, N8, N23, V25, I27, N41, V43, R45, D47, G62, T63, I64, H66, D68, H69, K70, N85, V86, M87, H89, S102, A103, T104, I105, M106, G120, L122, S138, P139<br>**CHAIN B:** E22, N23, W39, Y40, N41, D61, G62, H84, N85, M101, S102, A119, G120, T136, W153, N160, Y161, Y164, S167, Y168, Q171	**CHAIN A:** M1, I4, Y7, N8, V25, I27, N41, V42, V43, **R45**, **D47**, V48, T63, **I64**, **H66**, H69, M87, **H89**, S102, T104, M106, L122, S138,<br>**CHAIN B:** E22, N23, W39, **Y40**, N41, **Q60**, **D61**, **H84**, **N85**, **M101**, **S102**, T136, N160, **Y161**, Y164, Y167, **Y168, Q171**
	**Interface AC**	**CHAIN A:** M1, F19, A21, E22, **N23**, A24, S37, W39, **Y40**, **N41**, V42, V43, N58, I59, Q60, **D61**, G62, T63, I64, G83, H84, **N85**, V86, G100, **M101**, S102, A103, G118, A119, G120, A121, T136, G137, S138, K143, W153, K156, S157, N160, Y161, R163, **Y164**, K165, N166, S167, Y168, L169, N170, Q171,<br>**CHAIN C:** M1, A2, I3, I4, K5, P6, Y7, N8, G9, E22, N23, A24, V25, I26, I27, G28, D29, Y40, N41, V42, V43, I44, **R45**, G46, D47, V48, D61, G62, T63, I64, I65, H66, V67, D68, H69, K70, H84, N85, V86, M87, L88, H89, A90, S102, A103, T104, I105, M106, D107, G120, A121, L122, V123, T124, P125, S138, P139	**CHAIN A:** N23, **W39**, **Y40**, N41, Q60, **D61**, **H84**, N85, M101, S102, N160, **Y161**, **Y164**, S167, Y168, N170, Q171,<br>**CHAIN C:** I4, K5, Y7, N8, V25, I27, N41, V43, **R45**, **D47**, T63, **I64**, **H66**, H69, N85, V86, M87, H89, S102, T104, M106, D107, L122	**CHAIN A:** N23, **W39, Y40**, N41, **D61**, **H84**, N85, **M101**, S102, **A119,** N160, **Y161, Y164,** S167, **Y168**, Q171,<br>**CHAIN C:** A2, **I4**, Y7, N8, **V25**, **I27**, N41, **V43**, **R45**, D47, G62, **I64**, **H66**, H69, M87, **H89**, **T104, M106, L122**	**CHAIN A:** N23, W39, Y40, N41, D61, G62, H84, N85, M101, S102, A119, G120, T136, W153, N160, Y161, R163, Y164, S167, Y168, N170, Q171<br>**CHAIN C:** A2, I4, K5, P6, Y7, N8, V25, I27, N41, V43, R45, D47, V48, G62, I64, H66, D68, H69, K70, N85, V86, M87, H89, S102, A103, T104, I105, M106, D107, G120, L122, S138, P139	**CHAIN A:** E22, N23, W39, **Y40**, **N41**, Q60, **D61**, **H84**, N85, **M101**, **S102**, T136, N160, **Y161**, **Y164**, S167, **Y168**, Q171,<br>**CHAIN C:** I4, K5, **Y7**, V25, I27, **N41**, V42, V43, **R45**, **D47**, T63, I64, **H66**, H69, V86, M87, **H89**, **T104**, M106, L122, S138
	**Interface BC**	**CHAIN B:** M1, A2, I3, I4, K5, P6, Y7, N8, G9, K10, E22, N23, A24, V25, I26, I27, G28, Y40, N41, V42, V43, I44, **R45**, G46, D47, V48, N49, D61, G62, T63, **I64**, I65, H66, V67, D68, H69, K70, G83, H84, N85, V86, M87, L88, H89, A90, S102, A103, T104, I105, M106, D107, G120, A121, L122, V123, T124, P125, S138, P139,<br>**CHAIN C:** F19, A21, E22, **N23**, A24, S37, W39, **Y40**, N41, V42, V43, N58, I59, Q60, **D61**, G62, T63, V82, G83, **H84**, **N85**, V86, L98, G100, **M101**, S102, A103, G118, **A119**, G120, A121, T136, G137, S138, W153, K156, S157, Q159, N160, Y161, R163, Y164, K165, S167, Y168, L169, N170, Q171	**CHAIN B:**I4, K5, Y7, N8, V25, I27, N41, **V43**, **R45**, **D47**, V48, T63, **I64**, **H66**, D68, H69, K70, N85, V86, M87, L88, **H89**, S102, T104, I105, M106, L122,<br>**CHAIN C:** N23, **W39**, **Y40**, N41, **Q60**, **D61**, T63, **H84**, N85, M101, S102, W153, K156, N160, Y161, Y164, S167, Y168, Q171	**CHAIN B:**I4, P6, Y7, N8, **V25, I27**, N41, **V43**, **R45**, **D47**, V48, G62, **I64**, **H66**, H69, **M87**, **H89**, T104, **L122**<br>**CHAIN C:** N23, **W39**, **Y40**, N41, **D61**, G62, **H84**, **N85, M101, A119**, N160, **Y161**, Y164, S167, Y168, Q171	**CHAIN B:** A2, I4, K5, P6, Y7, N8, V25, I27, N41, V43, R45, D47, V48, G62, I64, H66, D68, H69, K70, N85, V86, M87, H89, S102, A103, T104, I105, M106, G120, L122, S138, P139<br>**CHAIN C:** N23, W39, Y40, N41, D61, G62, H84, N85, M101, S102, A119, G120, T136, G137, W153, K156, N160, Y161, Y164, S167, Y168, Q171	**CHAIN B:** I4, Y7, N8, V25, I27, N41, V42, V43, **R45**, **D47**, V48, T63, **I64**, H66, H69, N85, M87, **H89**, S102, T104, M106, L122, S138<br>**CHAIN C:** N23, W39, **Y40**, N41, **Q60**, **D61**, **H84**, **N85**, M101, S102, T136, N160, **Y161, Y164**, S167, Y168, Q171
γ-PmCA	**Interface AB**	**CHAIN A:** M1, A2, I3, I4, K5, P6, Y7, K8, G9, K10, Y11, P12, E22, N23, A24, V25, I26, I27, G28, Y40, N41, V42, V43, I44, **R45**, G46, D47, V48, N49, G62, T63, **I64**, I65, H66, V67, D68, H69, K70, H84, N85, V86, M87, L88, H89, S102, A103, T104, V105, M106, D107, V109, G120, A121, L122, V123, T124, P125, S138, P139,<br>**CHAIN B:** F19, A21, E22, N23, A24, V25, S37, W39, **Y40, N41**, V42, V43, N58, I59, Q60, **D61**, G62, T63, V82, G83, H84, **N85**, V86, G100, **M101**, S102, A103, G118, A119, G120, A121, T136, G137, S138, W153, K156, S157, Q159, N160, Y161, R163, **Y164**, K165, N166, S167, **Y168**, L169, N170, Q171, L172	**CHAIN A:** I3, I4, K5, Y7, K8, Y11, N23, V25, I27, N41, V42, **V43**, **R45**, **D47**, V48, **I64**, **H66**, V67, D68, H69, N85, V86, M87, L88, H89, S102, T104, V105, M106, L122, S138,<br>**CHAIN B:** N23, **W39, Y40**, N41, **Q60, D61, H84**, N85, M101, S102, W153, N160, **Y161, Y164,** S167, Y168, N170, Q171, L172	**CHAIN A:** **I4**, K5, P6, Y7, K8, **V25**, **I27**, N41, **V43**, **R45**, D47, **I64**, **H66**, H69, N85, **M87**, **H89, T104**, M106, **L122**<br>**CHAIN B:** N23, **W39**, **Y40**, N41, **D61**, **H84**, N85, **M101**, S102, **A119**, **Y161**, **Y164**, S167, **Y168**, N170, Q171, L172	**CHAIN A:** A2, I3, I4, K5, P6, Y7, K8, Y11, N23, V25, I27, N41, V43, R45, D47, V48, G62, I64, H66, V67, D68, H69, K70, N85, V86, M87, H89, S102, A103, T104, V105, M106, G120, L122, S138, P139<br>**CHAIN B:** N23, W39, Y40, N41, D61, G62, H84, N85, M101, S102, A119, G120, T136, W153, K156, N160, Y161, Y164, S167, Y168, N170, Q171, L172	**CHAIN A:**I4, K5, Y7, K8, V25, I27, N41, V42, V43, **R45**, **D47**, V48, T63, I64, **H66**, H69, N85, M87, **H89**, T104, M106, L122, S138<br>**CHAIN B:**N23, W39, **Y40**, N41, **Q60, D61, H84**, N85, M101, S102, T136, **Y161, Y164**, S167, Y168, N170, **Q171, L172**
	**Interface AC**	**CHAIN A:** F19, A21, E22, N23, A24, S37, W39, **Y40**, **N41**, V42, V43, N58, I59, Q60, **D61**, G62, T63, I64, G83, H84, **N85**, V86, G100, **M101**, S102, A103, I117, G118, A119, G120, A121, T136, G137, S138, K143, W153, K156, S157, N160, Y161, R163, **Y164**, K165, N166, S167, **Y168**, L169, N170, Q171, L172,<br>**CHAIN C:** M1, A2, I3, I4, K5, P6, Y7, K8, G9, P12, E22, N23, A24, V25, I26, I27, G28, D29, Y40, N41, V42, V43, I44, **R45**, G46, D47, V48, D61, G62, T63, **I64**, I65, H66, V67, D68, H69, K70, H84, N85, V86, M87, L88, H89, A90, C91, S102, A103, T104, V105, M106, D107, G120, A121, L122, V123, T124, P125, S138, P139	**CHAIN A:** N23, **W39, Y40**, N41, **D61, H84,** N85, M101, S102, W153, N160, **Y161, Y164,** S167, **Y168**, Q171, L172,<br>**CHAIN C:** M1, I3, I4, K5, Y7, K8, V25, I27, N41, V43, **R45, D47**, T63, **I64, H66**, D68, H69, N85, V86, M87, H89, S102, T104, V105, M106, L122	**CHAIN A:** N23, **W39**, **Y40**, N41, **D61**, **H84,** N85, **M101**, **S102, A119**, G120, **Y161, Y164,** S167, **Y168**, Q171, L172<br>**CHAIN C:** M1, **I4**, P6, Y7, **V25**, **I27,** N41, **V43**, **R45**, D47, **I64**, **H66,** N85, **M87**, **H89, T104**, M106, **L122**	**CHAIN A:** E22, N23, W39, Y40, N41, D61, G62, H84, N85, M101, S102, A119, G120, G137, W153, N160, Y161, R163, Y164, S167, Y168, Q171, L172<br>**CHAIN C:** M1, A2, I3, I4, K5, P6, Y7, K8, V25, I27, N41, V43, R45, D47, V48, G62, I64, H66, D68, H69, N85, M87, H89, S102, A103, T104, V105, M106, D107, L122, S138	**CHAIN A:** E22, N23, **W39**, **Y40**, **N41**, **Q60, D61, H84**, N85, **M101**, S102, T136, N160, **Y161**, **Y164**, S167, Y168, Q171<br>**CHAIN C:** A2, I3, K5, N23, V25, W39, Y40, N**41, V43, R45, D47**, D61, G62, **I64**, **H66**, V67, H84, N85, M87, **H89**, S102, T104, G120, T136
	**Interface BC**	**CHAIN B:** M1, A2, I3, I4, K5, P6, Y7, K8, G9, K10, Y11, P12, E22, N23, A24, V25, I26, I27, G28, Y40, N41, V42, V43, I44, **R45**, G46, D47, V48, D61, G62, T63, **I64**, I65, H66, V67, D68, H69, K70, G83, H84, N85, V86, M87, L88, H89, A90, M101, S102, A103, T104, V105, M106, D107, G120, A121, L122, V123, T124, P125, S138, P139,<br>**CHAIN C:** M1, F19, A21, E22, N23, A24, V25, S37, W39, **Y40**, N41, V42, V43, N58, I59, Q60, **D61**, G62, T63, V82, G83, H84, **N85**, V86, G100, **M101**, S102, A103, G118, **A119**, G120, A121, L122, T136, G137, S138, K143, W153, K156, S157, Q159, N160, Y161, R163, Y164, K165, N166, S167, **Y168**, L169, N170, Q171, **L172**	**CHAIN B:** I3, I4, K5, **Y7**, K8, V25, **I27**, N41, **V43**, **R45**, **D47**, T63, **I64**, **H66,** H69, N85, V86, M87, H89, S102, T104, V105, M106, L122,<br>**CHAIN C:** N23, **W39**, **Y40**, N41, **Q60, D61, H84**, N85, M101, S102, N160, **Y161**, **Y164**, S167, **Y168**, Q171, L172	**CHAIN B:** **I4**, K5, P6, **Y7**, K8, **V25**, **I27**, N41, **V43, R45, D47**, G62, **I64**, **H66**, **M87**, **H89**, **T104**, M106, **L122**<br>**CHAIN C:** N23, **W39, Y40,** N41, **D61,** **H84**, N85, **M101**, S102, A119, **Y161,** **Y164**, S167, **Y168,** Q171, L172	**CHAIN B:** A2, I3, I4, K5, P6, Y7, K8, E22, V25, I27, N41, V43, R45, D47, V48, G62, T63, I64, H66, D68, H69, K70, N85, V86, M87, H89, S102, T104, V105, M106, G120, L122, S138, P139<br>**CHAIN C:** N23, W39, Y40, N41, D61, G62, H84, N85, M101, S102, A119, G120, T136, W153, K156, N160, Y161, Y164, S167, Y168, N170, Q171, L172	**CHAIN B:** I4, Y7, K8, V25, **I27**, N41, V42, V43, **R45**, **D47**, V48, T63, I64, H66, N85, V86, M87, **H89**, S102, T104, M106, L122, S138<br>**CHAIN C:** N23, W39, **Y40**, N41, **Q60, D61**, H84, **N85**, M101, S102, T136, N160, **Y161,** Y164, S167, **Y168,** N170, **Q171**, L172
γ-TtCA	**Interface AB**	**CHAIN A:** S2, V3, Y4, R5, **F6**, E7, D8, K9, T10, P11, A12, V13, G22, A23, Y24, V25, V26, G27, A28, G40, A41, V42, V43, **R44**, G45, D46, L47, E48, G61, A62, V63, L64, H65, A66, D67, P68, G81, H82, R83, A84, V85, V86, H87, G88, V97, G98, M99, G100, A101, V102, V103, L104, N105, A117, G118, A119, V120, V121, P122, P123, G135, V136, P137<br>**CHAIN B:** F18, A20, P21, G22, A23, Y24, W38, **F39**, G40, A41, N57, V58, Q59, **D60**, G61, A62, V63, G81, H82, **R83**, A84, V85, G98, **M99**, G100, A101, V102, G116, A117, G118, A119, V120, L134, G135, V136, P137, N149, R152, Y153, A155, L156, A157, E158, R159, **Y160**, R161, K162, A163, **L164**, F165, P166, **V167**	**CHAIN A:** V3, **Y4**, R5, **F6, E7**, K9, T10, Y24, **V26**, V42, **R44**, **D46**, L47, **V63**, **H65**, R83, V85, H87, V102, L104, V120, V136,<br>**CHAIN B: W38, F39, Q59, D60, H82, R83,** M99, V136, R152, **Y153**, L156, R159, **Y160**, L164, F165, V167	**CHAIN A: V3, Y4,** R5, **F6,** E7, T10, P21, G22, **Y24**, **V26**, **V42, R44**, D46, L47, **V63, H65**, R83, **A84, V85**, H87, G100, **A101**, **V102, L104, V120**, V136,<br>**CHAIN B: W38**, **F39**, **D60, H82, R83, M99, A117, G118**, V136, R152, **Y153, L156**, R159, **Y160**, A163, **L164,** F165, P166, V167	**CHAIN A:** S2, V3, Y4, R5, F6, E7, K9, T10, P11, Y24, V26, G40, V42, R44, G45, D46, L47, G61, V63, H65, P68, R83, V85, H87, G100, A101, V102, L104, N105, V120, V136, P137,<br>**CHAIN B:** P21, G22, Y24, W38, F39, G40, D60, G61, H82, R83, M99, G100, A117, G118, A119, G135, V136, R152, Y153, L156, R159, Y160, A163, L164, F165, P166, V167	**CHAIN A:** S2, V3, **Y4**, R5, **F6, E7**, T10, **Y24**, V26, V42, **R44, D46**, L47, **V63**, **H65**, R83, **V85**, H87, V102, L104, V120, V136<br>**CHAIN B:** W38, **F39**, Q59, **D60**, **H82, R83, M99**,L134, V136 , R152 **, Y153 , L156 , R159 , Y160, L164,** F165, **V167**
	**Interface AC**	**CHAIN A:** S2, F18, A20, P21, G22, A23, Y24, S36, W38, **F39**, G40, A41, N57, V58, Q59, **D60**, G61, A62, G81, H82, **R83**, A84, V85, G98, **M99**, G100, A101, V102, G116, A117, G118, A119, V120, L134, G135, V136, N149, R152, Y153, A155, L156, A157, E158, R159, **Y160**, R161, K162, A163, L164, F165, P166, V167, A168, T169<br>**CHAIN C:** M1, S2, V3, **Y4**, R5, **F6**, E7, D8, K9, T10, P11, A12, V13, H14, P15, A17, I19, G22, A23, Y24, V25, V26, G27, A28, V29, G40, A41, **V42**, V43, **R44**, G45, D46, L47, E48, D60, G61, A62, V63, L64, H65, A66, D67, P68, G69, F70, H82, R83, A84, V85, V86, H87, G88, G98, M99, G100, A101, V102, V103, L104, N105, G118, A119, V120, V121, P122, P123, V136, P137	**CHAIN A: W38, F39, Q59, D60, H82, R83**, M99, V136, R152, **Y153, L156**, R159, **Y160**, L164, F165, V167, T169<br>**CHAIN C:** M1, S2, V3, **Y4**, **R5**, **F6**, E7, T10, Y24, **V26**, V42, **R44**, **D46**, L47, **V63**, **H65**, D67, R83, V85, H87, V102, L104, V120, V136	**CHAIN A:** P21, G22, **W38**, **F39, D60**, **H82, R83, M99**, A117, G118, R152, **Y153, L156**, R159, **Y160,** A163, **L164**, F165, P166, V167, A168, T169<br>**CHAIN C:** M1, S2, **V3, Y4**, R5, **F6,** E7, **Y24, V26, V42, R44, D46**, L47, **V63**, **H65**, D67, P68, R83, **V85**, **H87**, G100, **A101, V102, L104, V120**	**CHAIN A:** P21, G22, Y24, W38, F39, G40, D60, G61, H82, R83, M99, G100, A117, G118, A119, L134, G135, V136, N149, R152, Y153, L156, R159, Y160, K162, A163, L164, F165, P166, V167, A168, T169<br>**CHAIN C:** M1, S2, V3, Y4, R5, F6, E7, T10, P11, V13, I19, Y24, V26, G40, V42, R44, D46, L47, G61, V63, H65, D67, P68, R83, V85, H87, G100, A101, V102, L104, N105, V120, V136, P137	**CHAIN A:** W38, **F39**, Q59, **D60**, **H82, R83**, M99, L134, V136 , R152 **, Y153 , L156 , R159 , Y160, L164,** F165, V167, T169,<br>**CHAIN C:** S2, V3, **Y4**, R5, **F6**, **E7, Y24**, V26, V42, **R44, D46, L47, V63, H65**, D67, R83, V85, H87, V102, L104, V120, V136
	**Interface BC**	**CHAIN B:** V3, Y4, R5, **F6**, E7, D8, K9, T10, P11, V13, I19, G22, A23, Y24, V25, V26, G27, A28, G40, A41, **V42**, V43, **R44**, G45, D46, L47, E48, G61, A62, V63, L64, H65, A66, D67, P68, H82, R83, A84, V85, V86, H87, G88, G98, M99, G100, A101, V102, V103, L104, N105, G118, A119, V120, V121, P122, P123, G135, V136, P137,<br>**CHAIN C:** M1, F18, A20, P21, G22, A23, Y24, S36, W38, **F39**, G40, A41, N57, V58, Q59, **D60**, G61, A62, G81, H82, **R83**, A84, V85, G98, **M99**, G100, A101, V102, G116, A117, G118, A119, V120, L134, G135, V136, N149, R152, Y153, A155, L156, A157, E158, R159, **Y160**, R161, K162, A163, **L164**, F165, P166, V167, A168	**CHAIN B:** V3, **Y4**, **R5, F6**, E7, T10, Y24, **V26**, V42, **R44**, **D46**, L47, **V63**, **H65**, R83, V85, H87, V102, L104, V120, V136,<br>**CHAIN C:** Y24, **W38, F39, Q59, D60, H82, R83**, M99, V136, **Y153**, **L156**, R159, **Y160**, L164, F165, V167	**CHAIN B:** V3, **Y4**, R5, **F6**, E7, **Y24, V26, V42, R44**, D46, L47, **V63**, **H65**, R83, **V85**, **H87**, G100, **V102, L104, V120**, V136,<br>**CHAIN C:** P21, G22, **W38, F39, D60, H82, R83, M99**, A117, **G118**, V136, **Y153, L156,** R159, **Y160**, A163, **L164**, F165, P166, V167	**CHAIN B:** V3, Y4, R5, F6, E7, T10, Y24, V26, G40, V42, R44, G45, D46, L47, G61, V63, H65, D67, R83, A84, V85, H87, G100, V102, L104, V120, V136, P137<br>**CHAIN C:** P21, G22, Y24, W38, F39, G40, D60, G61, H82, R83, M99, G100, A117, G118, A119, G135, V136, R152, Y153, L156, R159, Y160, A163, L164, F165, P166, V167, A168	**CHAIN B:** V3, **Y4, R5, F6, E7**, **Y24**, V26, V42, **R44, D46,** L47, **V63, H65**, R83, V85, H87, V102, L104, V120, V136<br>**CHAIN C:** W38, **F39, Q59, D60, H82, R83**, M99, L134, V136, R152, **Y153, L156, R159, Y160, L164**, F165, **V167**
γ-TtkCA	**Interface AB**	**CHAIN A:** I2, A3, Y4, K5, G6, V7, G20, S21, F22, V23, I24, G25, W36, F37, N38, T39, V40, V41, **R42**, G43, D44, V45, N46, I56, Q57, D58, L59, T60, **M61**, V62, H63, V64, T65, T66, A67, K68, I79, G80, H81, R82, A83, M84, V85, H86, G99, A100, I101, I102, L103, D104, G117, S118, L119, V120, T121, S135, P136,<br>**CHAIN B:** I2, F16, A18, E19, G20, S21, F22, S34, I35, W36, **F37, N38**, T39, V40, N55, I56, Q57, **D58, L59**, T60, M61, T78, I79, G80, H81, **R82**, A83, M84, L95, G97, **M98**, G99, A100, I101, A115, **A116,** G117, S118, L119, M133, G134, S135, L154, H157, Y158, L161, A162, S164, Y165, D168	**CHAIN A:** I2, Y4, K5, **F22**, I24, N38, T39, **V40**, **R42**, D44, V45, L59, T60, **M61**, **H63**, V64, R82, **M84, H86**, I101, L103, L119,<br>**CHAIN B:** E19, **W36, F37**, N38, **Q57, D58, L59, H81, R82**, M98, M133, H157, Y158, L161, Y165, D168	**CHAIN A: Y4, F22, I24**, N38, **V40, R42**, D44, V45, L59, **T60, M61, H63**, R82, **A83, M84, H86, I101, L103, L119**, P136,<br>**CHAIN B:** E19, G20, **W36, F37**, N38, **D58, L59, H81, R82, M98**, G99, **A116**, G117, M133, H157, **Y158,** L161, Y165	**CHAIN A:** I2, Y4, K5, G6, F22, I24, N38, V40, R42, D44, V45, L59, T60, M61, H63, T66, A67, R82, A83, M84, H86, G99, A100, I101, L103, L119, S135, P136<br>**CHAIN B:** E19, G20, F22, W36, F37, N38, Q57, D58, L59, H81, R82, M98, G99, I101, A116, G117, M133, G134, H157, Y158, L161, Y165, D168	**CHAIN A:** I2, Y4, **F22**, I24, N38, T39, **V40**, **R42**, D44, V45, L59, T60, M61, **H63**, V64, R82, M84, H86, **I101**, L103, **L119,** S135<br>**CHAIN B:** E19, W36, **F37**, N38, Q57, **D58, T59, H81, R82**, M98, M133, H157, **Y158**, L161, Y165, D168
	**Interface AC**	**CHAIN A:** I2, F16, A18, E19, G20, S21, F22, S34, W36, **F37, N38**, T39, V40, N55, I56, Q57, **D58**, **L59**, T60, M61, T78, I79, G80, **H81**, **R82**, A83, M84, G97, **M98**, G99, A100, L113, A115, A116, G117, S118, L119, M133, G134, S135, R153, L154, H157, Y158, L161, A162, S164, Y165, D168,<br>**CHAIN C:** M1, I2, A3, Y4, K5, G6, V7, G20, S21, F22, V23, I24, G25, W36, F37, N38, T39, V40, V41, **R42**, G43, D44, V45, N46, Q57, D58, L59, T60, M61, V62, H63, V64, T65, T66, A67, K68, G80, H81, R82, A83, M84, V85, H86, M98, G99, A100, I101, I102, L103, D104, A116, G117, S118, **L119**, V120, T121, S135, P136	**CHAIN A:** E19, **W36, F37**, N38, **Q57, D58, L59, H81, R82**, M98, H157, **Y158**, L161, Y165, D168,<br>**CHAIN C:** Y4, K5, **F22**, I24, N38, T39, **V40**, **R42, D44**, V45, L59, T60, **M61, H63**, R82, M84, H86, I101, L103, L119	**CHAIN A:** E19, G20, **W36, F37, N38, D58, L59, H81**, R82, **M98**, G99, **A116**, G117, H157, **Y158, L161**, **Y165**, D168,<br>**CHAIN C:** Y4, G6, **F22, I24**, N38, **V40, R42**, D44, V45, L59, T60, **M61, H63**, R82, **M84, H86**, G99, **I101, L119**	**CHAIN A:** E19, G20, F22, W36, F37, N38, D58, L59, H81, R82, M98, G99, A116, G117, M133, L154, H157, Y158, L161, S164, Y165, D168<br>**CHAIN C:** I2, Y4, K5, G6, V7, F22, I24, N38, V40, R42, D44, V45, L59, T60, M61, H63, T66, A67, R82, A83, M84, H86, G99, A100, I101, L103, D104, G117, L119, S135, P136	**CHAIN A:** E19, W36, **F37**, N38, **Q57, D58, L59, H81, R82**, M98, H157, **Y158**, L161, **Y165**, D168,<br>**CHAIN C: Y4, F22**, I24, N38, T39, V40, **R42, D44**, V45, L59, T60, M61, **H63**, R82, M84, **H86, I101, L119**, S135
	**Interface BC**	**CHAIN B:** M1, I2, A3, Y4, K5, G6, V7, G20, S21, F22, V23, I24, G25, I35, W36, F37, N38, T39, V40, V41, **R42**, G43, D44, V45, N46, Q57, D58, L59, T60, **M61**, V62, H63, V64, T65, T66, A67, G80, H81, R82, A83, M84, V85, H86, G87, G99, A100, I101, I102, L103, D104, G117, S118, L119, V120, T121, Q122, S135, P136,<br>**CHAIN C:** M1, I2, F16, A18, E19, G20, S21, F22, S34, W36, **F37**, **N38**, T39, V40, N55, I56, Q57, **D58**, **L59**, T60, M61, T78, I79, G80, H81, **R82**, A83, M84, L95, G97, **M98**, G99, A100, L113, A115, **A116**, G117, S118, M133, G134, S135, E147, R153, L154, H157, Y158, L161, A162, S164, Y165, D168	**CHAIN B:** Y4, K5, V7, F22, I24, N38, T39, **V40**, **R42, D44**, V45, L59, T60, **M61, H63,** R82, M84, H86, I101, L103, L119, S135,<br>**CHAIN C:** E19, W36, **F37**, N38, **Q57, D58, L59, H81, R82,** M98, H157, **Y158,** L161, Y165, D168	**CHAIN B:**Y4, **F22, I24**, N38, **V40, R42**, D44, V45, L59, **M61, H63**, R82, **M84, H86, L119**<br>**CHAIN C:** E19, **W36, F37**, N38, **D58, L59, H81**, R82, **M98, A116**, G117, H157, **Y158, L161, Y165**	**CHAIN B:** Y4, K5, G6, V7, G20, F22, I24, N38, V40, R42, D44, V45, L59, T60, M61, H63, A67, R82, A83, M84, H86, I101, L103, D104, L119, S135, P136<br>**CHAIN C:** M1, E19, G20, F22, W36, F37, N38, D58, L59, H81, R82, M98, G99, A116, G117, M133, H157, Y158, L161, Y165, D168	**CHAIN B:** I2, **Y4, F22**, I24, N38, T39, V40, **R42, D44**, V45, T59, T60, M61, **H63**, R82, M84, **H86**, **I101**, L103, L119, S135,<br>**CHAIN C:** E19, W36, **F37, N38**, Q57, D**58, T59, H81, R82**, M98, M133, H157, **Y158**, L161, **Y165**, D168

Table S2: Interface characteristics of the trimeric structures of γ-CAs.

Carbonic anhydrase	Hydrogen bonds		Salt bridges		Buried surface area/ Å^2	Total surface area/ Å^2	% BSA
	PDBePISA	PIC	PDBePISA	PIC	PDBePISA		
γ-PhCA	18	40	10	5	5,155	24,780	20.9
γ-PmCA	12	38	8	6	5,353	25,327	21.1
γ-TtCA	41	60	26	9	6,846	23,823	28.7
γ-TtkCA	15	44	9	5	4,749	23,574	20.1
